# Supplementary material for: Weekends-Off Lenvatinib for Unresectable Hepatocellular Carcinoma Improves Therapeutic Response and Tolerability Toward Adverse Events
Source: Cancers (Basel). 2020 Apr 19;12(4):1010. doi: 10.3390/cancers12041010 (PMC7226076; doi:10.3390/cancers12041010)
Supplement: Supplementary file 1 [file cancers-12-01010-s001.pdf]

Supplementary Materials

Weekends-off Lenvatinib for Unresectable Hepatocellular Carcinoma Improves Therapeutic Response and Tolerability Toward Adverse Events

Hideki Iwamoto, Hiroyuki Suzuki, Shigeo Shimose, Takashi Niizeki, Masahito Nakano, Tomotake Shirono, Shusuke Okamura, Yu Noda, Naoki Kamachi, Toru Nakamura, Atsutaka Masuda, Takahiko Sakaue, Toshimitsu Tanaka, Dan Nakano, Miwa Sakai, Taizo Yamaguchi, Ryoko Kuromatsu, Hironori Koga and Takuji Torimura

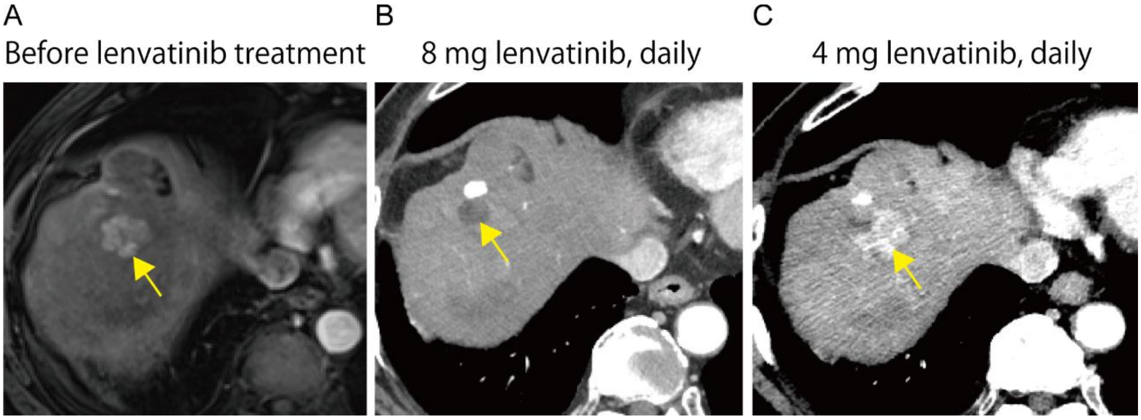

Figure S1. Representative computed tomography images during lenvatinib treatment. (**A**) before treatment (the arrow shows enhanced lesion). (**B**) Treated with 8 mg lenvatinib, daily (the arrow shows the lesion with disappeared enhancement). (**C**) After dose reduction from 8 mg to 4 mg (the arrows show the lesions that has regrown and has been re-enhanced).

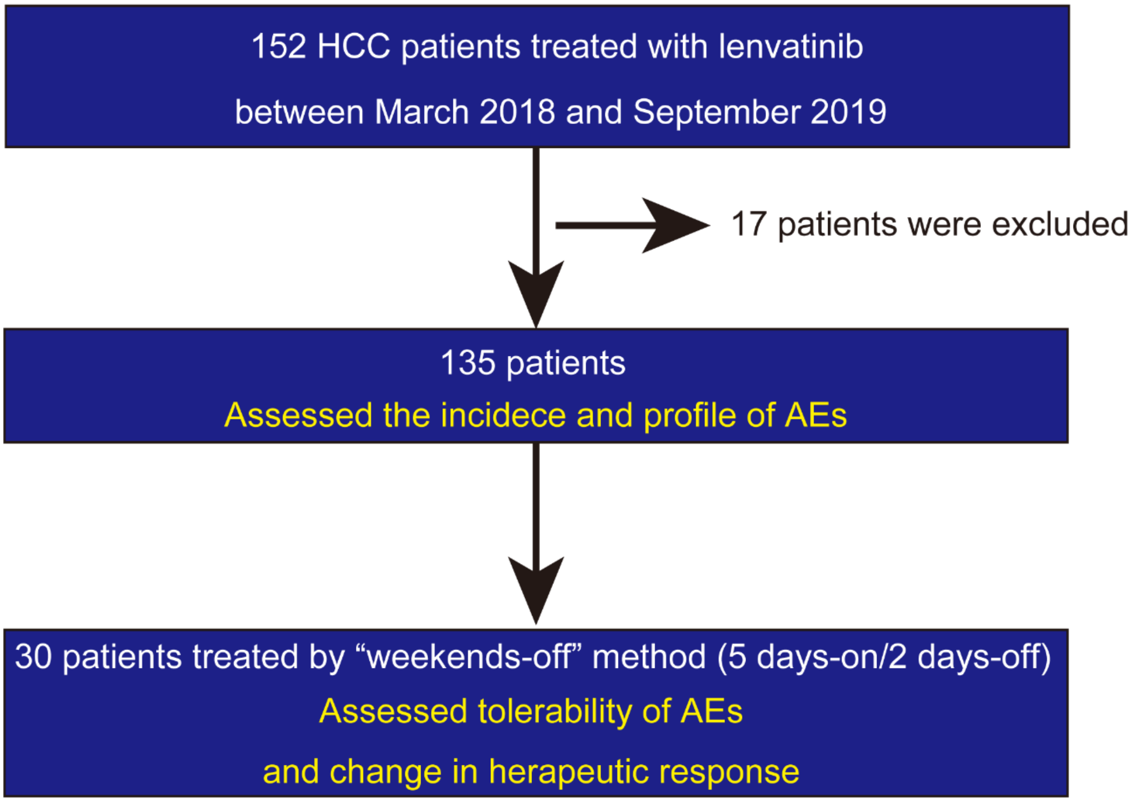

Figure S2. Patient inclusion flowchart. AEs, adverse events; HCC, hepatocellular carcinoma.

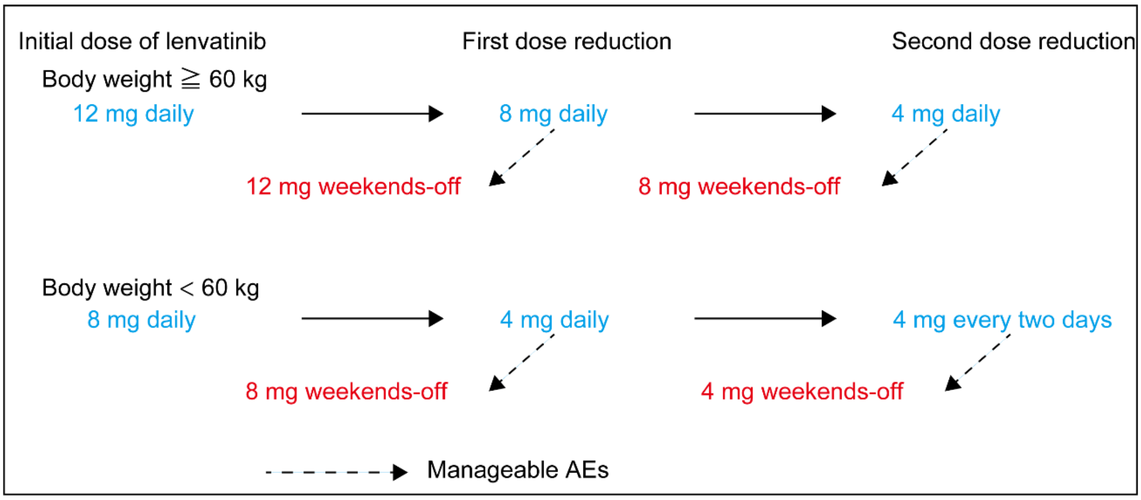

Figure S3. Protocol for dose reduction and weekends-off administration of lenvatinib. The initial dose of lenvatinib was 12 mg once daily for patients weighing ≧ 60 kg and 8 mg once daily for those weighing < 60 kg. In the patients who received 12 mg, the first reduction was to 8 mg and the second was to 4 mg. In the patients who received 8 mg, the first reduction was to 4 mg once daily and the second was to 4 mg divided in two administrations a day. The dose reduction was maintained until the AEs were improved to grade 1 or 2. If the reduced dose was acceptable, it was increased when switched to the weekends-off administration, which meant a cycle of five consecutive days-on/two consecutive days-off at the original dose. AEs: Adverse events

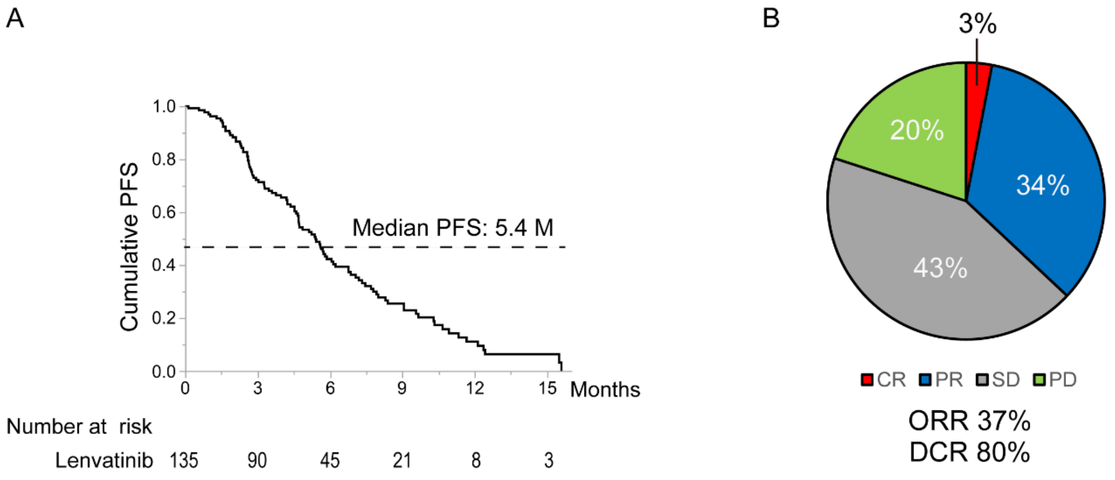

Figure S4. PFS and treatment response in the overall cohort (n=135 patients). (**A**) PFS. The median PFS was 5.4 months. (**B**) Therapeutic response. The proportion of patients with a CR, PR, SD and PD was 3%, 34%, 43%, and 20%, respectively; the ORR was 37%; and the DCR was 80%. Abbreviations: PFS, progression-free survival; CR, complete response; PR, partial response; PD, progressive disease; SD, stable disease ORR, objective response rate; DCR, disease control rate. ;.

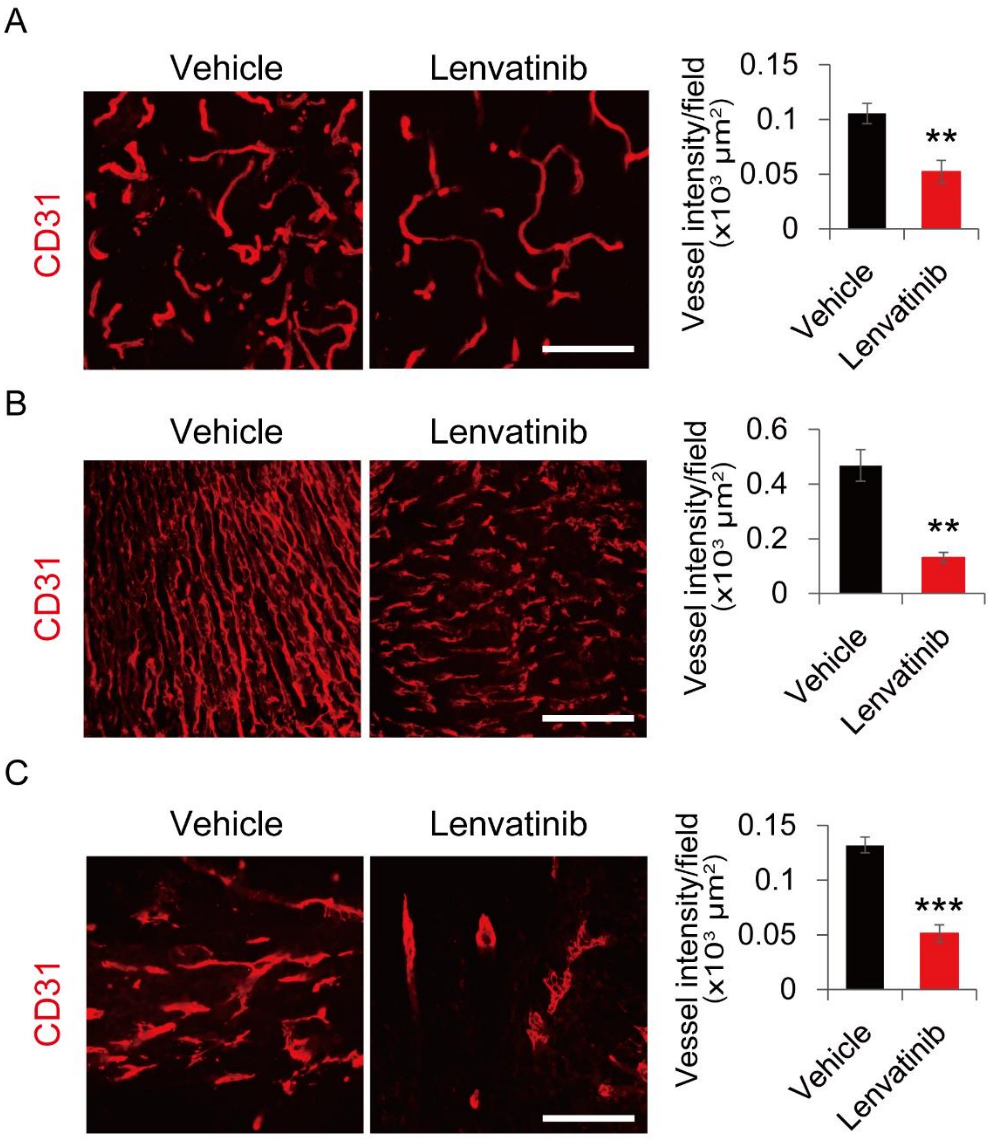

Figure S5. Assessment of vascular changes in the thyroid and adrenal glands and the tumor in the mouse hepatoma orthotopic model. (**A**) Vessels of the thyroid in the vehicle and the lenvatinib treatment group. Quantification of CD31+ vessels of thyroid (6 random field from three independent samples per group). (**B**) Vessels of the adrenal gland in the vehicle and the lenvatinib treatment groups. Quantification of CD31+ vessels of the adrenal gland (6 random field from three independent samples per group). (**C**) Tumor vessels in the vehicle and the lenvatinib treatment groups. Quantification of CD31+ tumor vessels (6 random field from three independent tumor samples per group). Bar represents 200 μm. $^{**}p < 0.01$, $^{***}p < 0.001$. Data are presented as means ± SEM.

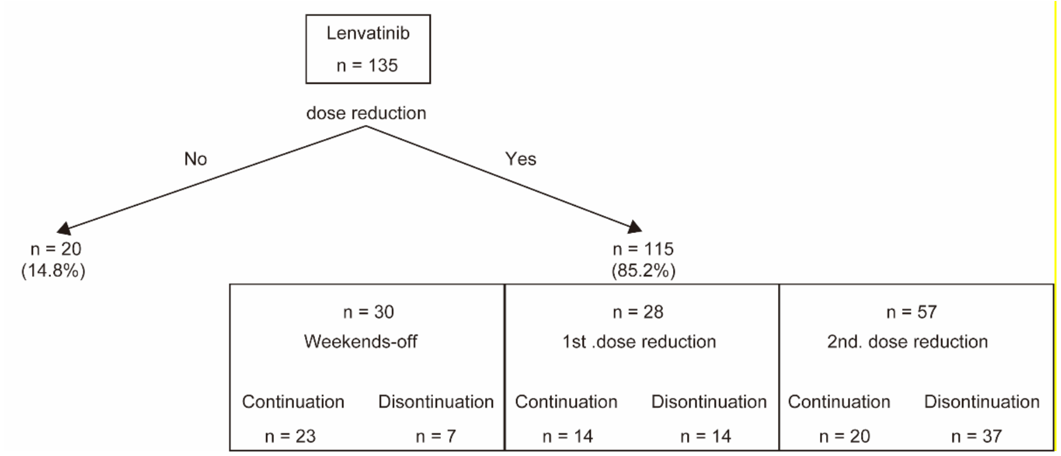

Figure S6. Overall flowchart of the patients treated with lenvatinib in this study. Of the 135 patients, 20 patients did not need any dose reduction of lenvatinib, while the other 115 patients needed a dose reduction. Of the 115 patients, 30 patients received the "weekends-off" protocol, 28 patients received the first reduced dose of lenvatinib, and 57 patients the second reduced dose of lenvatinib.

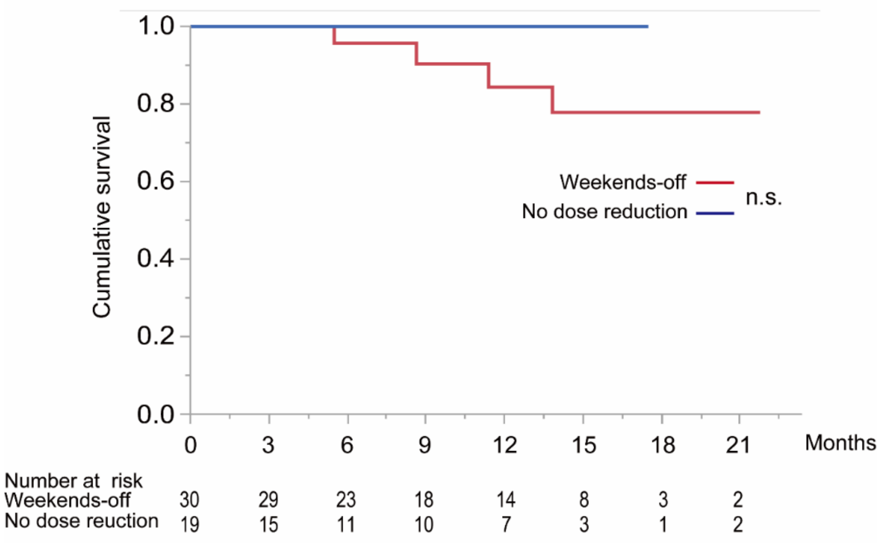

Figure S7. Comparison of the OS between the "weekends-off" protocol group and the no dose reduction group. No dose reduction group included patients who did not need any dose reduction of lenvatinib (14.8% of all 135 patients treated with lenvatinib in this study). There was no significant difference between the two groups.

Table S1. Baseline clinicodemographic and tumor characteristics of the 118 patients who evaluated the function of thyroid among 135 patients.

**Clinicodemographic characteristics of 118 patients**	**Value**
Age (years)	73 (44–89)
Sex<br>Male/Female	100/18
Etiology<br>HBV/HCV/non-B, non-C	24/54/40
Child-Pugh score<br>5/6/7	81/29/8
ALBI grade<br>1/2	47/71
Body weight<br>Less than 60 kg/over 60 kg	54/64
Tumor characteristics	
BCLC stage<br>A/B/C	2/69/47
TNM stage<br>II/III/IVA/IVB	6/64/5/43
Tumor size (mm)	31 (10–170)
AFP* (ng/mL)	18.2 (1.5–118,560)
DCP** (mAU/mL)	165.5 (11.5–524,068)
Lenvatinib	
Initial dose (mg)<br>4/8/12	6/74/38
Thyroid function	
Hypothyroidism<br>Yes/no	14/104
Subclinical hypothyroidism<br>Yes/no	71/47

* Alpha-fetoprotein ; ** Des-gamma carboxyprothrombin.

Table S2. Baseline clinicodemographic and tumor characteristics of the 31 patients who evaluated the function of adrenal gland among 135 patients.

**Clinicodemographic characteristics of 31 patients**	**Value**
Age (years)	77 (51–89)
Sex	
Male/Female	23/8
Etiology	
HBV*/HCV**/non-B, non-C	4/13/14
Child-Pugh score	
5/6	22/9
ALBI grade***	
1/2	11/20
Body weight	
Less than 60 kg/over 60 kg	14/17
Tumor characteristics	
BCLC stage	
A/B/C	1/18/12
TNM stage	
II/III/IVA/IVB	1/18/1/11
Tumor size (mm)	24 (10–170)
AFP† (ng/mL)	18.2 (1.5–118,560)
DCP†† (mAU/mL)	171 (12–179,531)
Lenvatinib	
Initial dose (mg)	
4/8/12	1/19/11
Adrenal function	
Hypoadrenocorticism	
Yes/no	0 /31
Elevation of ACTH†††	
Yes/no	12/19

* Hepatitis B virus; ** Hepatitis C virus; *** Albumin-bilirubin grade; † Alpha-fetoprotein; †† Des-gamma carboxyprothrombin; ††† Adrenocorticotropic hormone.

Table S3. Baseline clinical and tumor characteristics of the patients treated with lenvatinib "weekends-off".

**Clinical characteristics of 30 patients**	**value**
Age (years)	77 (53–88)
Sex<br>Male/Female	26/4
Etiology<br>HBV*/HCV**/non-B, non-C	7/13/10
Child-Pugh score<br>5/6	25/5
ALBI grade***<br>1/2	17/13
Body weight<br>Less than 60 kg/over 60 kg	13/17
Tumor characteristics	
BCLC stage<br>A/B/C	0/14/16
TNM stage<br>II/III/IVA/IVB	14/2/14
Tumor size (mm)	27 (10-111)
AFP† (ng/mL)	18.2 (1.5–43,480)
DCP†† (mAU/mL)	805.5 (11.5–179,531)
Lenvatinib	
Initial dose (mg)<br>4/8/12	1/15/14

* Hepatitis B virus; ** Hepatitis C virus; *** Albumin-bilirubin grade; † Alpha-fetoprotein; †† Des-gamma carboxyprothrombin.

Table S4. AEs which led to a reduction in lenvatinib dosage in 30 patients.

AEs	n
Fatigue	10
Proteinuria	6
Anorexia	5
Hand-foot syndrome	4
Diarrhea	4
Thrombocytopenia	3
Vomiting	1
Gingival hemorrhage	1
Hoarseness	1
Ascites	1
Elevation of ALT	1

AE: adverse event.

© 2020 by the authors. Licensee MDPI, Basel, Switzerland. This article is an open access article distributed under the terms and conditions of the Creative Commons Attribution (CC BY) license (http://creativecommons.org/licenses/by/4.0/).
